# New progress of tuberculosis scar carcinoma

**DOI:** 10.1007/s10555-023-10128-9

**Published:** 2023-08-16

**Authors:** Wenwen Sun, Yujin Liu, Lishu Zhao, Hao Wang, Li Ye, Xinyue Liu, Kandi Xu, Yu Chen, Lin Fan

**Affiliations:** 1 Department of Tuberculosis Department Shanghai Pulmonary Hospital, Tongji University Medical School Cancer Institute, Tongji University School of Medicine, No 507 Zhengmin Road, Shanghai, 200433 China; 2 Tongji University, No 1239 Siping Road, Shanghai, 200433 China; 3 Department of Medical Oncology, Shanghai Pulmonary Hospital, Tongji University Medical School Cancer Institute, Tongji University School of Medicine, No 507 Zhengmin Road, Shanghai, 200433 China; 4 Department of Spine Surgery, Second Affiliated Hospital of Naval Medical University (Changzheng Hospital), Shanghai, China

**Keywords:** Lung cancer, Tuberculosis, Scar carcinoma, Scar tissue, Inflammation

## Abstract

It has been demonstrated that scar tissue and fibrosis may increase the likelihood of developing malignancies. Specifically, scar tissue has been linked to the occurrence and progression of lung cancer (LC), though the precise mechanisms necessitate further research for explanation. Lung scarring can stem from various causes, with carcinogenesis on scarring lesions in pulmonary tuberculosis (PTB) being the most frequent (accounting for approximately 75% of cases). Notably, having previously cured, PTB is the second most common risk factor for LC after smoking, with approximately 3% of PTB patients experiencing LC as a secondary condition. This essay will delve into the mechanisms, treatment, and prognosis of tuberculosis scar carcinoma (TSC).

## Introduction

Tuberculosis (TB) is a highly infectious disease caused by *Mycobacterium tuberculosis* (MTB) and primarily affects the lungs, resulting in high morbidity and mortality rates. According to the World Health Organization (WHO) statistics for 2020, the global proportion of people with TB is 56 per 100,000 population, with 10 million new cases and approximately 1.5 million deaths attributed to the disease each year [1]. Lung cancer (LC) is a significant health concern with low survival rates, and several factors like smoking, air pollution, diet, inflammation, and prior respiratory illnesses can trigger its development [2]. Research has indicated that TB may be a contributing risk factor in the development and progression of LC, with a coexistence rate of approximately 2% [3]. Due to the complex interactions between these diseases, the precise mechanisms underlying the relationship between TB and LC remain incompletely understood. Nevertheless, prevailing theories suggest the involvement of inflammatory stimulation, immune function, DNA damage, and scar calcification [4]. Given the diverse disease manifestations and complex interactions, research remains ongoing to fully understand the relationship between TB and LC. Additionally, the scarring of pulmonary tuberculosis (PTB) lesions is a crucial risk factor for LC development [5].

The concept of scar carcinoma (SC) was first introduced by Friedrich Scholar in 1939, defining it as a group of malignancies that arise from scars around the lungs [6]. Since then, numerous studies have been conducted to investigate the mechanisms by which lung scarring can trigger the development of LC. However, these studies have mainly consisted of single or small patient case reports, and more large-scale population-based epidemiological and data analyses are required. SC is a relatively rare condition that mostly affects males and typically involves the upper lobe of the lung. The histopathological type of this condition is usually adenocarcinoma [7].

## Historical studies

The diagnostic criteria for scar included lesions that did not involve the main bronchi, continuous hyalinized scars on pathological examination, and initial scar tissue that did not contain cancer cells [6]. In 1972, Freant et al. conducted a retrospective study of 119 SC patients from 1961 to 1972 to investigate the relationship between scar tissue and cancer. A large number of histopathological examinations revealed the presence of elastic fibers and anthrax pigments in the lesion, while tumor masses could be found in some dense hyaline connective tissue or adjacent areas. In some cases, cancer tissues of varying degrees of differentiation were seen around scars, from epithelial hyperplasia to metaplasia to overt carcinoma [8]. Auerbach et al. conducted a comprehensive retrospective study, analyzing 1186 cases of LC patients from over 7000 autopsy cases between 1955 and 1975, revealing a total of 82 cases of SC. The outcomes showed that peripheral LCs accounted for 15% of SCs, and 45% of these cases were caused by scar tissue. Among the SC cases, 72% were adenocarcinomas, with 75% of cancers located in the upper lobe, and more than half of the cases were related to infarcts caused by scar tissue [9]. The study conducted by Luders and Themel investigated the etiology of pulmonary scars, in which they stated that pulmonary scars were mostly due to PTB, and TB scars played a crucial role in SC formation [10]. Another research work by Brett et al. reviewed 917 patients diagnosed with LC from 2013 to 2017 through imaging at a South African hospital, of which 268 had lung scars or diffuse fibrosis, suggesting a strong association between lung scar and primary LC [11].

Based on the survey, it was observed that individuals with a history of PTB had twice the risk of developing LC as compared to the general population [12]. PTB patients often experience inflammation due to recurrent tissue damage and repair, with some underlying infections causing inflammation that results in the production of inflammatory factors, such as interleukin (IL) and tumor necrosis factor (TNF), during their long-term treatment. As a result of this inflammation, acute phase proteins are produced, leading to tissue fibrosis and ultimately formation of scar tissue [13]. Several large-scale studies have been conducted to investigate the relationship between TB and LC. A study conducted by Engles et al. on 42,422 farmers in Xuanwei, China, from 1976 to 1996 involved a series of standardized questionnaires and follow-up visits. It was found that the mortality rate of LC in patients with PTB was significantly greater than in those without PTB (25 vs. 3.1 per 1000 person-years) [14]. Zheng et al. have suggested that LC may develop as a consequence of PTB on the same side of the lung [15]. The PLCO trial followed 66,863 cancer-free individuals aged 55 to 74 years for 12 years, and those with lung scars on chest radiography had 1.8 times higher risk of developing LC than controls (HR = 1.8; CI 1.4–2.4) [16]. Additionally, Everatt et al. observed a 3.5-fold increase in LC incidence in 21,986 PTB patients from Lithuania compared to the non-PTB population, with 477 of the PTB patients developing LC during the final follow-up [17]. Su et al. conducted a retrospective analysis of 11,522 subjects with latent tuberculosis infection (LTBI) along with 46,088 matched subjects, revealing that PTB contacts had a 2.7 times higher risk of LC, indicating a higher incidence of LC in the LTBI group [18]. Furthermore, TB has also been associated with the occurrence of secondary LC. Ho et al. conducted a large national cohort study that followed 1,936,512 individuals with or without TB and observed a 1.67-fold increase in secondary LC risk in TB patients compared to the non-TB population of primary cancer [19]. Related studies are presented in Table 1.

**Table 1:** Epidemiological study of tuberculosis, pulmonary scars, and lung cancer

S. no	Study cases	Methods	Results	Conclusions	Ref
1	119 patients with lung adenocarcinoma	Retrospective study	Abundant elastic fibers identified within the lesion	SC is closely related to peripheral adenocarcinoma	8
2	1186 LC autopsy cases, including 82 cases of SC	Retrospective study	15% of these SC was associated with peripheral LCs, while 45% of them resulted from scar tissue	Scar tissue is an important factor in causing LC	9
3	917 LC patients from a hospital in South Africa	Retrospective study	Of these, 268 had pulmonary scarring or diffuse fibrosis	Pulmonary scarring is strongly associated with primary LC	11
4	42,422 farmers in Xuanwei, China	Retrospective study	The mortality rate of LC in patients with PTB was significantly higher than that in patients without PTB	PTB is a risk factor for LC	14
5	66863 subjects aged 55–74 years without cancer	Retrospective cohort study	Patients with lung scars on chest radiography had a higher risk of LC than the control group (HR = 1.8; CI1.4, 2.4)	Pulmonary scarring is a risk factor for LC	16
6	21,986 PTB patients from Lithuania	Retrospective study	477 PTB patients developed LC, which was 3.5 times the incidence in the population without PTB	PTB is a risk factor for LC	17
7	11,522 LTBI subjects and 46,088 matched subjects	Retrospective cohort study	The incidence of LC was 2.7 times higher in TB contacts than in the matched control group	LTBI is a risk factor for LC	18
8	Nationwide screening of 1,936,512 subjects	Retrospective cohort study	Compared to the non-TB population, patients with TB had a 1.67-fold increased risk of secondary LC	TB is a risk factor for secondary LC	19

## Inflammatory mechanisms of lung cancer caused by tuberculosis scar

The process of fibrotic scarring in PTB results from repeated tissue damage and repair. This process is associated with mechanisms of long-term pulmonary inflammation that increase the risk of LC [20]. Initially, reactive oxygen species (ROS) produced at the lesion promote inflammation and stimulate the production of cytokines such as TNF, IL-1, and IL-6. These cytokines, in turn, stimulate the expression of cyclooxygenase (COX) -2, IL-2, C-reactive protein (CRP), and nuclear factor kappa B (NF-κB), thus further intensifying the inflammatory response. Inflammatory factors can also induce the synthesis of P-selectin and E-selectin, which in turn activate endothelial cells to produce vascular cell adhesion molecule (VCAM) and intercellular adhesion molecule (ICAM). These processes activate leukocytes and allow them to accumulate in the lesion, while free radicals produced by activated leukocytes can cause DNA damage in the lesion cell [21]. In the presence of inflammatory cytokines, nitric oxide (NO) is produced and can oxidize DNA and even damage some DNA repair proteins [22]. Inflammatory factors can also affect genome integrity by inhibiting cytochrome P-450 or glutathione S-transferase isoenzymes [22]. Therefore, ROS-induced inflammatory responses can promote DNA damage, thereby increasing the endogenous carcinogenic risk.

**Fig. 1:**
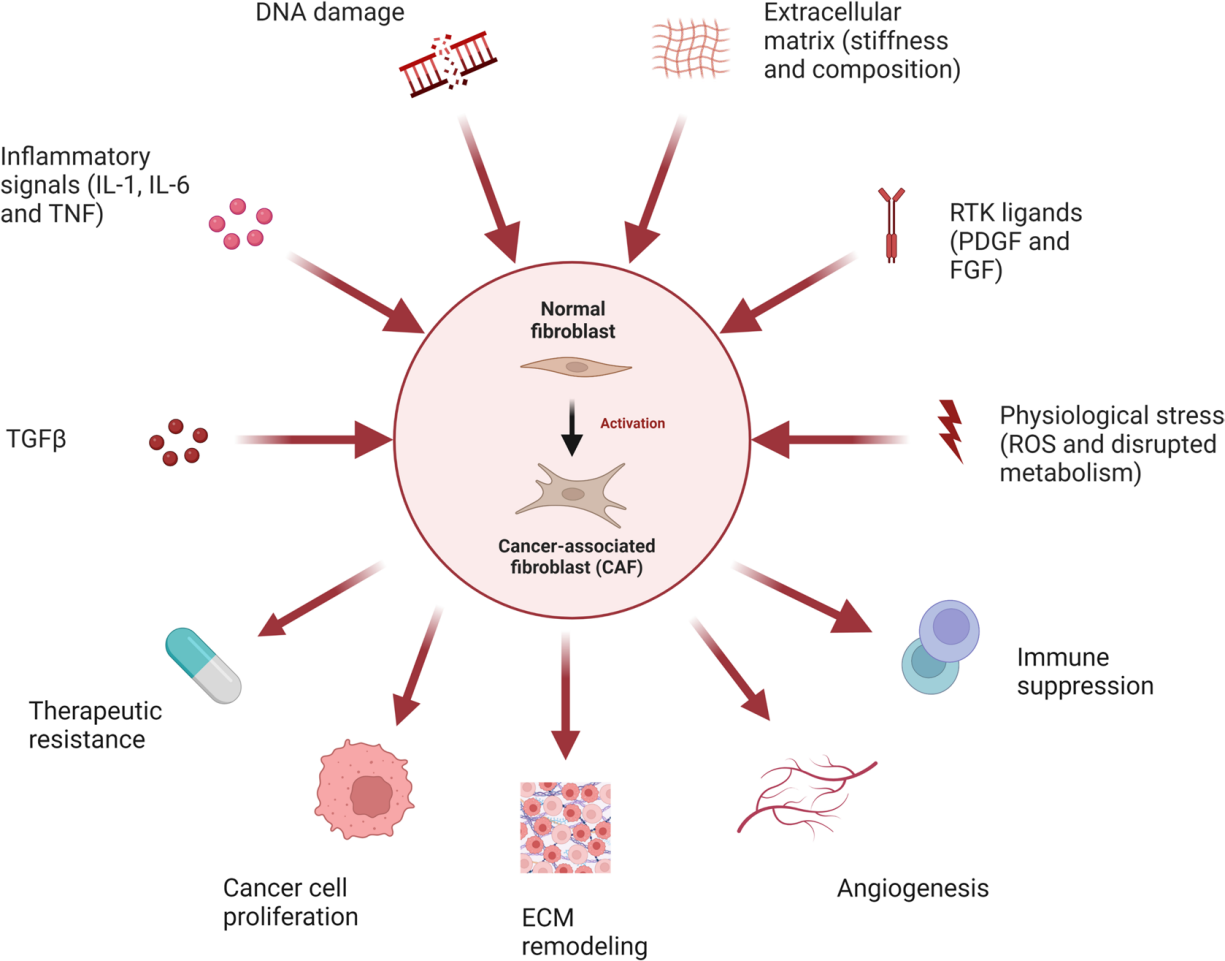
Mechanisms of cancer-associated fibroblast activation and its effect on tumor microenvironment

**Fig. 2:**
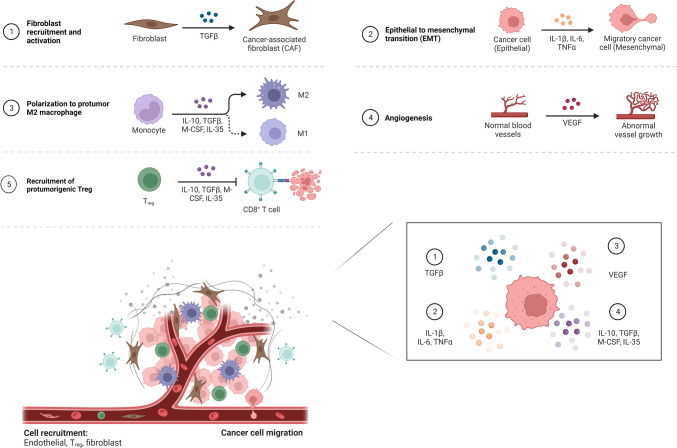
Scar inflammation mechanisms affect the tumor microenvironment and promote the development of cancer

TNF, IL-1, and IL-6 can stimulate the production of angiogenic factors, such as vascular endothelial growth factor (VEGF), and transforming growth factor (TGF) -β1, produced by inflammatory macrophages [22]. TGF-β, TNF-α, and IL-related factors can induce lung remodeling, promote residual fibrosis of the lesion, and trigger the production of cancer-associated fibroblasts (CAF). As depicted in Fig. 1, CAF activation is linked with the activation of ROS, inflammatory cascade processes, and genetic damage. CAF and angiogenic factors are both dense and indispensable components of the tumor microenvironment [23]. TGF-β and IL-related factors, which are produced by inflammation-stimulated macrophages, can induce the production of heat shock protein (HSP47), promote collagen synthesis by lung fibroblasts, and eventually form scar tissue. During a chronic inflammatory response, persistent cascades lead to enlarged inflammation, causing persistent DNA damage, apoptosis, and residual fibrosis, which may eventually lead to gene mutations activating oncogenes and slowly evolving into cancer cells. An increase in fibrosis obstructs the flow of blood and lymphoid tissue through the scar tissue, further promoting the growth of cancer cells [24], as shown in Fig. 2. This mechanism can result in the inability of normal cells to adapt, leading to their death and their replacement by variant cells that can adapt to this abnormal growth environment, which are likely to be malignant cells.

The presence of inflammatory monocytes (IMs) is a known cause of scar tissue formation and can also be found in pathological tissue specimens. This is particularly true for squamous cell carcinoma, which is often characterized by a massive infiltration of IMs. Inhibition of the accumulation of IMs can be an effective drug target, potentially leading to inhibited tumor metastasis. It is worth noting that IMs express factor XIIIA at high levels, which in turn promotes fibrin cross-linking and creates scaffolds to aid in the invasion and metastasis of squamous cell carcinoma cells [25]. Chemokine ligand 2 (CCL-2) is responsible for the recruitment of IMs, and elevated levels of CCL-2 have also been found in patients with PTB [26]. Furthermore, IMs have the ability to differentiate into dendritic cells or tumor-associated macrophages (TAMs), both of which can play an important role in promoting tumor growth [27].

## Treatment and prognosis of tuberculosis scar carcinoma

Early detection is the key to treating TSC. Clinical studies suggest that a comprehensive approach using multiple treatment methods, such as surgery, radiotherapy, anti-tumor drug chemotherapy, immunotherapy, targeted therapy, traditional Chinese medicine treatment, and intervention, should be selected based on the specific pathological type, TNM stage, lesion location, size, extent, and overall patient health to maximize efficacy. The ultimate goal is to alleviate patient pain, improve quality of life, and prolong overall survival (OS) [28].

### Surgical treatment of tuberculosis scar carcinoma

In cases of severe fibrosis, radiotherapy is not recommended. Additionally, there are no systematic studies of chemotherapy regimens for SC. As a result, radical surgery is the preferred method for treating localized lesions with no serious complications. During surgery, it is important to thoroughly dissect intrapulmonary and mediastinal lymph nodes and perform radical surgery. However, due to its high malignancy, lymphatic or hematogenous metastasis and the prognosis are generally poor [28].

### Chemotherapy for tuberculosis scar carcinoma

When it comes to treating SC, there are a variety of drug options available. In addition to traditional anti-tumor drugs, there have been large-scale studies exploring the effectiveness of drugs such as steroids, ulinastatin, sivelestat sodium, and macrolides in the treatment process, although they have not yet been clinically confirmed [29]. Hypoxia-inducible factor (HIF) plays a role in promoting LC metastasis and is associated with inflammation-related signaling pathways. As a result, HIF inhibitors are being widely studied as novel anticancer agents [30, 31]. Other drugs, such as pirfenidone, inhibit the expression of fibroblast growth factor (bFGF), TGF-β, CTGF, and TIMP-1, reducing the production of type I and type III collagen fibers. Pirfenidone also inhibits inflammatory mediators to varying degrees, exerting its anti-inflammatory effects [32, 33]. However, pirfenidone alone is not effective at inhibiting cancer cells. Combining it with cisplatin has been shown to achieve better efficacy because pirfenidone can enhance the efficacy of cisplatin by inhibiting the production of scars to change the tumor microenvironment and increase drug permeability [32]. Paclitaxel has also been demonstrated to be effective in inhibiting the growth of SC cells by inducing apoptosis and hindering the cell cycle [34]. Additionally, oral LY2109761, proposed by Wei et al., has been shown to reduce TGF-β1-induced collagen production, which inhibits scar fibrosis progression. This provides new ideas for the treatment of patients with early TSC. However, it is important to note that while these drugs show promise, further clinical testing may still be required [35].

### Immunotherapy for tuberculosis scar carcinoma

Immunotherapy is a novel treatment modality that has been actively researched in recent years. Of particular interest are immune checkpoint inhibitors (ICI) that have shown promise for cancer therapy such as programmed cell death protein-1 (PD-1) and its ligand (PD-L1) inhibitors. Clinically, their use is based on the level of PD-L1 expression. For example, advanced non-small cell lung cancer (NSCLC) with PD-L1 expression greater than 50% can be treated with the PD-1 antibody, Keytruda (pembrolizumab), as a monotherapy with a response rate of approximately 40% [36]. According to a first-line monotherapy trial based on relevant research, the survival rate of NSCLC patients was compared between PD-(L)1 antibody therapy and platinum-based first-line chemotherapy. The final results showed that pembrolizumab significantly extended patients’ survival time, especially for those with higher PD-L1 expression [37]. PD-1 and cytotoxic T-lymphocyte–associated protein 4 (CTLA-4) are complementary co-inhibitory receptors; so, according to the latest relevant clinical trials, the combination of PD-1 and CTLA-4 inhibitors is expected to improve patient efficacy compared to monotherapy [38]. In addition to PD-(L)1 antibody therapy, different checkpoint inhibitor combinations, with or without chemotherapy, may also improve patients’ survival rates. Therefore, it is particularly important to select appropriate targets for individualized treatment according to patients’ conditions. The efficacy of immunotherapy for SC is influenced by several factors, such as cancer type, stage, and timing of diagnosis [39].

### Prognosis of tuberculosis scar carcinoma

The prognosis of TSC is still debated, with some scholars suggesting that the prognosis seems favorable in the LC population (with a 5-year survival rate between 5/6 and 3/7) [40]. However, Freant et al. reported a 5-year survival rate of only 5% after SC surgery [8]. Studies have indicated that that the prognosis of pulmonary scar carcinoma (PSC) is primarily linked to the cancer cell type and lesion stage, and not necessarily to the scar itself. SC adenocarcinomas typically invade blood vessels and lymph nodes, leading to distant metastasis and a poor prognosis. Carroll proposed that scar tissue proliferation may hinder lymphatic and blood drainage, while carcinogens accumulate at the scar, promoting the spread of its blood vessels and lymph nodes [41]. Freant et al. also found that SC lymph nodes are often heavily involved, and the presence or absence of lymph node metastasis is an important prognostic indicator [8]. Early lymph node metastasis may be a unique feature of SC. Therefore, clinicians should remain vigilant for the possibility of SC, monitor lesions closely, and pay extra attention to patients with a history of pulmonary PTB to achieve early diagnosis and treatment. In conclusion, patients with PTB scars are at an increased risk of LC and require close monitoring and follow-up.

## Conclusion

Studies in history have confirmed the relationship between TB and LC, but the mechanisms that govern the interaction between these two diseases are not yet fully understood. TB infection can cause chronic inflammation and plays an integral role in scarring. While most current research on TSC comes from published case reports or series, additional TSC samples and data studies are necessary to better comprehend their biological behavior and to develop potential new treatments. A potentially difficult issue with SC diagnosis is that histological examination is performed long after a precipitating event, which may lead to scar formation. Since determining the timing of the initial injury is challenging, evidence of scarring prior to SC has been a point of debate for decades. To better understand how scar problems lead to cancer development, it is essential to track the scar’s development over time and observe its eventual transition into cancer through imaging. The diagnosis and treatment of TSC remain a challenge in various clinical settings.

## Data Availability

The data used in this study were obtained from publicly accessible academic databases such as PubMed and Web of Science. These data were obtained through legitimate channels and comply with the terms and conditions of these databases. In this paper, we have cited relevant data obtained from PubMed and Web of Science to support our research findings and perspectives. We have taken appropriate measures to ensure the accuracy and reliability of the data. It is important to emphasize that the use of these data is in accordance with the regulations of the respective databases and adheres to applicable laws and ethical guidelines. We commit to citing these data appropriately and providing correct citation information in the references. Please note that this study may also include third-party materials from other sources, such as figures, images, and quotations. We have made reasonable efforts to ensure that the use of these third-party materials complies with copyright laws and fair use principles. Unless otherwise stated, these materials are also subject to the data availability statement of this paper. If you have any questions or need further information regarding the availability of specific data, please contact the data providers such as PubMed and Web of Science directly.
